# Kurt Semm: A laparoscopic crusader

**DOI:** 10.4103/0972-9941.37197

**Published:** 2007

**Authors:** O P Sudrania

**Affiliations:** Grant Medical College, Mumbai; MGM Medical College, Kishanganj, India

Sir,

It is indeed heartening to read an account on the biography of Kurt Semm - the oceanic personality who is rightfully acclaimed worldwide as the “Father of Gynecological Laparoscopy”.

However, Bhattacharya mentions that he preferred the term ‘pelviscopy’ for operative laparoscopy. He used it to differentiate between gynecological laparoscopy and procedures that other specialties performed for upper abdominal screening and liver biopsies.” Though true verbatim, but it was only a sequel to the prevailing bad reputation against the word ‘laparoscopy’ which had profound legal and insurance implication beyond its diagnostic purposes during those days. The period between 1940s to early 1960s was regarded as the period of accidents. I quote Kurt Semm himself, “At the beginning of laparoscopy in Gynecology there were serious accidents (period of serious accidents). The result of this was that at the beginning of the 1960s laparoscopy in Gynecology was absolutely forbidden.”[1] Please mark his words - ‘absolutely forbidden.’ Therefore changing name from laparoscopy to pelviscopy helped him to overcome the legal and social stigmata so deeply enrooted those days. This in turn helped him to convince the insurance companies to pay more fees for a more prolonged procedure.[2]

Bhattacharya further mentions - Raoul Palmer, was an American Surgeon. This is a repetition of the same mistake which Annette Tuffs had committed in her obituary on Kurt Semm. However, this was corrected by Herbert E Reiss in his rapid response.[3] I quote him, “Kurt Semm's obituarist names Palmer as an American Surgeon.[4] Raoul Palmer in fact was a great French Gynecologist who taught most of the world's tubal surgeons before the advent of IVF. His work place, life and love were Paris.”

A further correction to the same effect was published by the Editorial Team[4] as: ‘Correction for Tuffs: Obituary of Kurt Semm’. It reads, ‘In this obituary of Kurt Semm supplied by Annette Tuffs (16 August, p 397),[5] Raoul Palmer was wrongly referred to as an American Surgeon, he was in fact French’. Again Bhattacharya's observation, “and finally laparoscopic-assisted vaginal hysterectomy (nowadays termed as Cervical intrafascial Semm hysterectomy)” deserves further comment for which I shall like to reproduce Kurt Semm himself once again, “All these technical developments combined with a change of heart among the general surgeons allowed me to realize my dream in pelviscopic surgery: the complete pelviscopic hysterectomy. On September 7, 1991, I performed the first C. I. S. H. (Classic Intrafascial S. E. M. M. (Serrated Edged Macro Morcelator) Hysterectomy).”[6] In this technique, what he called C. I. S. H, ‘C’ stand for Classic instead of Cervical and Semm is S. E. M. M. - which should not be erroneously construed for Kurt Semm's surname by the unwary.
